# Birth seasonality and risk of autism spectrum disorder

**DOI:** 10.1007/s10654-019-00506-5

**Published:** 2019-03-20

**Authors:** Brian K. Lee, Raz Gross, Richard W. Francis, Håkan Karlsson, Diana E. Schendel, Andre Sourander, Abraham Reichenberg, Erik T. Parner, Mady Hornig, Amit Yaniv, Helen Leonard, Sven Sandin

**Affiliations:** 1 Department of Epidemiology and Biostatistics, A.J. Drexel Autism Institute, Drexel University School of Public Health, Philadelphia, PA USA; 2 Department of Epidemiology and Preventive Medicine, Sackler Faculty of Medicine, Tel Aviv University, Tel Aviv, Israel; 3 Division of Psychiatry, Chaim Sheba Medical Center, Tel Hashomer, Ramat Gan, Israel; 4 Telethon Kids Institute, The University of Western Australia, West Perth, WA Australia; 5 Department of Neuroscience, Karolinska Institute, Stockholm, Sweden; 6 Department of Public Health, University of Aarhus, Aarhus, Denmark; 7 Department of Economics and Business, University of Aaarhus, Aarhus, Denmark; 8 University Hospital of Turku and Department of Child Psychiatry, University of Turku, Turku, Finland; 9 Department of Psychiatry, New York State Psychiatric Institute, College of Physicians and Surgeons of Columbia University, New York, NY USA; 10 Department of Psychiatry and Department of Preventive Medicine, Icahn School of Medicine at Mount Sinai, New York, NY USA; 11 Seaver Autism Center for Research and Treatment at Mount Sinai, New York, NY USA; 12 Department of Epidemiology and Center for Infection and Immunity, Mailman School of Public Health, Columbia University, New York, NY USA; 13 Sackler Faculty of Medicine, Tel Aviv University and the Arrow Project for Junior Investigators, Sheba Medical Center, Tel Hashomer, Tel Aviv, Israel; 14 Department of Medical Epidemiology and Biostatistics, Karolinska Institutet, Stockholm, Sweden

**Keywords:** Autism, Seasonality, Epidemiology, Empirical mode decomposition

## Abstract

Season of birth has been hypothesized to be a risk factor for autism spectrum disorder (ASD). However, the evidence has been mixed and limited due to methodological challenges. We examine ASD birth trends for 5,464,628 births across 5 countries. ASD birth prevalence data were obtained from the International Collaboration for Autism Registry Epidemiology database, including children born in Denmark, Finland, Norway, Sweden, and Western Australia. Empirical mode decomposition and cosinor modeling were used to assess seasonality. We show seasonal variation in ASD births for the countries of Finland and Sweden. There was a modest increase in risk for children born in the fall and a modest decrease in risk for children born in the spring. Solar radiation levels around conception and the postnatal period were inversely correlated with seasonal trends in ASD risk. In the first multinational study of birth seasonality of ASD, there was evidence supporting the presence of seasonal trends in Finland and Sweden. The observations that risk was highest for fall births (i.e., conceived in the winter) and lowest for spring births (i.e., conceived in the summer), and sunlight levels during critical neurodevelopmental periods explained much of the seasonal trends, are consistent with the hypothesis that a seasonally fluctuating risk factor may influence risk of ASD.

**Electronic supplementary material:**

The online version of this article (10.1007/s10654-019-00506-5) contains supplementary material, which is available to authorized users.

## Introduction

ASD is a set of heterogeneous complex neurodevelopmental conditions, characterized by early-onset difficulties in social communication and unusually restricted, repetitive behavior and interest. Even though genetic factors explain much of the variation in ASD risk [1], environmental factors acting during the prenatal, perinatal, and postnatal periods also influence risk [2]. The changing of the seasons is associated with multiple environmental factors relevant to fetal development including reproduction, nutrition, infections, and chemical exposures [3–7]. The possibility of seasonality influencing risk of ASD was first raised in the 1980s [8]. However, studies of seasonality in ASD have reported mixed findings, such as an increased risk among children born in March [9]; excesses in other months [10]; or no seasonal trends at all [11, 12]. Inconsistent findings may be due to a number of factors, including differences in populations; geographic regions; lack of control for trends in ASD surveillance and ascertainment; and small samples.

The aim of the present analysis was to apply rigorous parametric and non-parametric statistical techniques to detect patterns of seasonality. We also aimed to assess whether such seasonality was consistent with observed trends in sunlight. Given the importance of vitamin D for proper neurodevelopment [13, 14] and the seasonal variability of sunlight, solar radiation is one factor that may help explain any observed seasonal trends.

## Methods

**Fig. 1:**
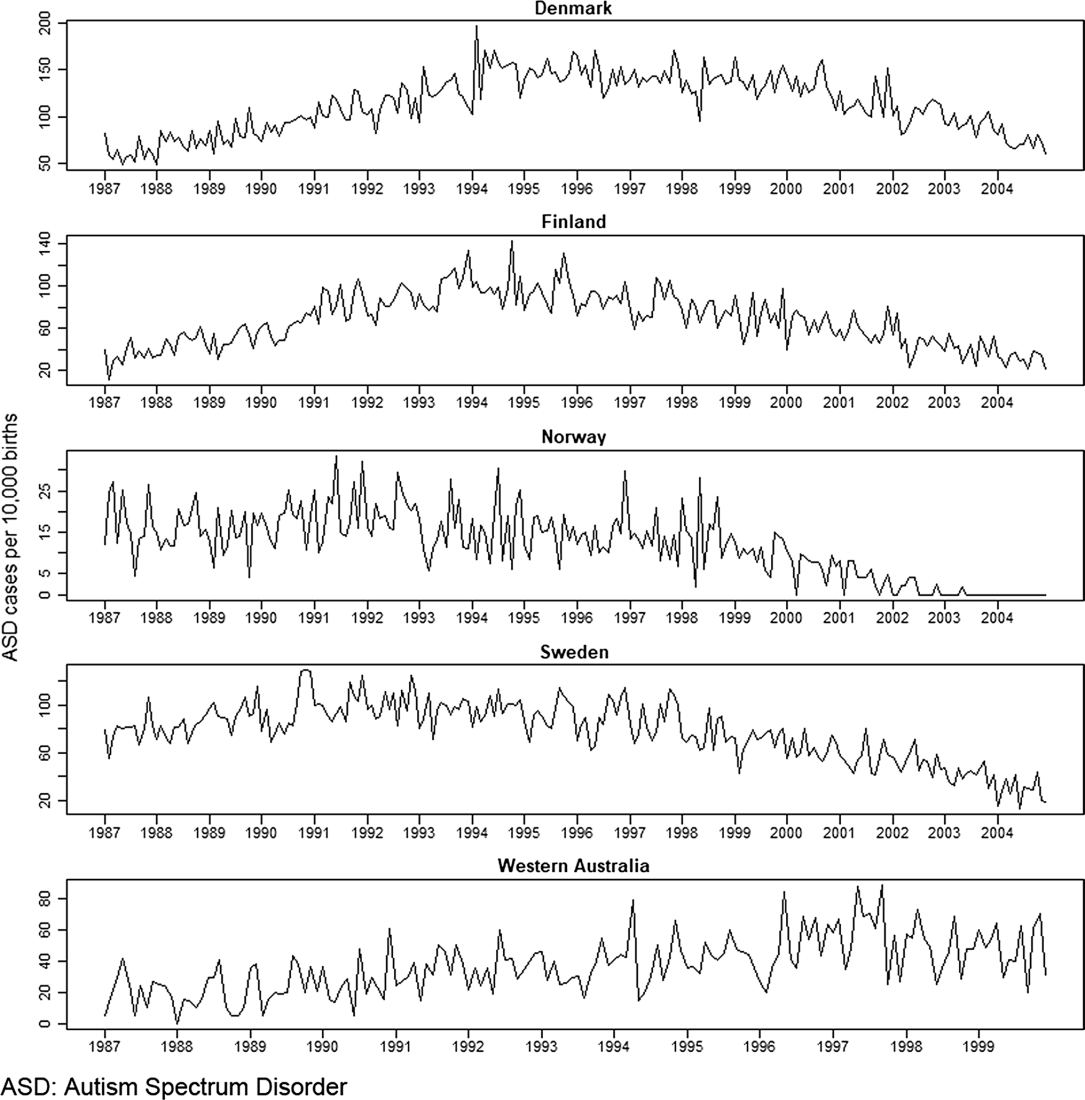
ASD prevalence time series (cases per 10,000) by country and birth month

Analysis of seasonal trends in ASD risk is complex because non-etiological calendar trends exist in ASD prevalence data. In particular, an inverse-U shape is seen in the prevalence rates by month of birth in each country (Fig. 1). The upward-sloped component of the inverse-U corresponds with the commonly reported increase in ASD prevalence over the years. Evidence suggests that most of this increase is probably due to non-etiological reasons, such as increases in awareness and changes in diagnostic criteria and reporting practices [15–17]. The downward-sloped component is an artifact of shorter length of follow-up: the less time a participant is followed, the less opportunity there is for a diagnosis of ASD, so children born more recently will have a lower probability of diagnosis. Thus, analyses need to account for these trends. The following analyses were applied to each country. First, the relative odds of ASD for different months of birth were examined in logistic regression models with adjustment for calendar time. Second, empirical mode decomposition (EMD) was used to remove calendar trends and decompose the ASD prevalence time series into component signals. Third, the component signals were analyzed for seasonality and compared against data regarding solar radiation.

ASD birth prevalence data were obtained from the International Collaboration for Autism Registry Epidemiology (iCARE), a multinational research consortium promoting autism research. Our analysis included children born in Denmark (N = 1,172,516 born 1987–2004 with follow-up through 2009); Finland (N = 1,087,827 born 1987–2004 with follow-up through 2009); Norway (N = 1,057,578 born 1987–2004 with follow-up through 2006); Sweden (N = 1,841,192 born 1987–2004 with follow-up through 2009); and Western Australia (N = 305,515 born 1987–1999 with follow-up through 2004) [18]. Ethical approvals, with waivers for informed consent, were obtained for each site. Case identification and validation, registry reporting, and data harmonization across sites is described elsewhere [19].

Analyses were performed using R 3.4.1 [20]. Logistic regression using generalized additive models in the R package *mgcv* was used to calculate odds ratios (ORs) and 95% two-sided confidence intervals. Indicator variables were used for month of birth, with the month of January as the Ref. [21]. Penalized regression splines were used to adjust for birth year [22].

EMD is a non-parametric tool to decompose non-linear and non-stationary time series into a finite number of component signals called intrinsic mode functions (IMFs) through an adaptive algorithm [23]. Previous use of EMD in epidemiology studies include analyses of dengue, depression, and hepatitis B and C [24–26]. The IMFs are computed by first defining two cubic spline functions as interpolations from the local maxima and the local minima. IMFs satisfy the following criteria: the number of extrema and zero crossings differ by at most one; and at any point, the local average is zero. These functions are averaged, and the mean is then subtracted from the original data. If the remainder satisfies IMF criteria, then the process is stopped. Otherwise, the remainder is treated as a new time series and the above steps iterated. We used EMD to decompose the ASD prevalence time series for each country into component IMFs for further analysis (Supplement Figures 1–5). Because the data exhibited mode-mixing of multiple sine components due to signal intermittency, ensemble EMD was implemented. The basic premise of ensemble EMD is that a small amount of white noise is added to the data. EMD was then applied with resulting IMFs defined as the mean of an ensemble of trials. White noise of 10% of the standard deviation of the original time series was added and the ensemble mean of 100 iterations was calculated. [27] The IMFs were assessed for statistical significance using permutation testing in order to determine whether they were different from random noise. The ensemble EMD and permutation testing were implemented using R code by Xie et al. [28] IMFs with signals beyond permutation testing-defined 99% confidence limits were then modeled using cosinor modeling in order to confirm seasonality. Cosinor regression models are a flexible method often used for studies of seasonal variation [29]. Models had the following form:

$$IMF(t) = \beta_{0} + \beta_{1} \times \cos \frac{2\pi t}{T} + \beta_{2} \sin \frac{2\pi t}{T}$$

where *T *= length of time of one period. *T* was set to 365 days in order to confirm the IMF data fit with a hypothesized seasonal model and *t *= the underlying time-scale variable, i.e. the number of days since Jan 1, 1987.

IMFs consistent with seasonal variation were then cross-correlated against incident solar radiation to determine the lagged time with which the largest correlations in the hypothesized direction were seen. The lagged sunlight data were then input into regression models to determine how much of the variance in the ASD seasonal IMFs they explained.

Solar radiation data were derived from satellite observations from the NASA Prediction of Worldwide Energy Resource [30]. Average monthly solar radiation (specifically: average insolation on a horizontal surface, MJ/m^2^/day) for the capital cities were calculated. Solar radiation and EMD residuals were standardized by their respective means and standard deviations to arrange the plots on the same z-score scale.

## Results

The sample consisted of 5,464,628 live born children, 37,734 with a recorded ASD diagnosis. ASD prevalence rates for each country are provided in Supplement Table 1. Analysis of ASD risk with reference to January showed that for Finland and Sweden, there were multiple months in the latter part of the year for which excess ASD risk was detected (Table 1). For Finland, there was 14–21% increased risk of ASD for the birth months of July, October, and December as compared with January. Similarly, for Sweden, there was 13–25% increased risk of ASD for the birth months of July, September, October, November, and December. In Denmark, there was an 11% increased risk in September; in Norway, a 26% decreased risk in February; and no differences for any month for Western Australia.

**Table 1:** Relative odds and 95% confidence intervals of ASD by birth month with respect to January

	Denmark	Finland	Norway	Sweden	Western Australia
Jan	Ref.	Ref.	Ref.	Ref.	Ref.
Feb	1.09 (1.00, 1.18)	1.00 (0.89, 1.13)	**0.74 (0.56, 0.99)**	1.01 (0.93, 1.10)	0.98 (0.73, 1.32)
Mar	0.99 (0.91, 1.08)	0.99 (0.88, 1.11)	0.93 (0.72, 1.21)	1.01 (0.93, 1.09)	0.94 (0.70, 1.26)
Apr	1.00 (0.91, 1.08)	1.07 (0.95, 1.20)	0.81 (0.62, 1.07)	0.99 (0.91, 1.07)	1.16 (0.87, 1.54)
May	1.02 (0.94, 1.11)	0.99 (0.88, 1.11)	1.04 (0.81, 1.35)	1.08 (1.00, 1.17)	0.98 (0.73, 1.32)
Jun	1.07 (0.98, 1.16)	1.01 (0.90, 1.13)	1.04 (0.80, 1.35)	1.02 (0.94, 1.11)	0.91 (0.67, 1.23)
Jul	1.04 (0.95, 1.13)	**1.14 (1.02, 1.27)**	1.06 (0.82, 1.37)	**1.13 (1.04, 1.22)**	1.03 (0.77, 1.37)
Aug	1.01 (0.93, 1.10)	1.10 (0.98, 1.23)	0.92 (0.70, 1.21)	1.05 (0.97, 1.14)	1.22 (0.92, 1.61)
Sep	**1.11 (1.02, 1.21)**	1.12 (1.00, 1.25)	1.02 (0.78, 1.33)	**1.16 (1.07, 1.26)**	1.1 (0.82, 1.45)
Oct	1.07 (0.98, 1.17)	**1.21 (1.08, 1.35)**	0.99 (0.76, 1.30)	**1.19 (1.09, 1.29)**	0.94 (0.70, 1.26)
Nov	1.06 (0.98, 1.16)	1.11 (0.99, 1.25)	1.03 (0.78, 1.35)	**1.24 (1.14, 1.34)**	1.15 (0.87, 1.54)
Dec	1.03 (0.95, 1.13)	**1.18 (1.05, 1.32)**	1.18 (0.91, 1.53)	**1.25 (1.15, 1.35)**	1.00 (0.74, 1.34)

Bold values indicate* p* < 0.05

**Fig. 2:**
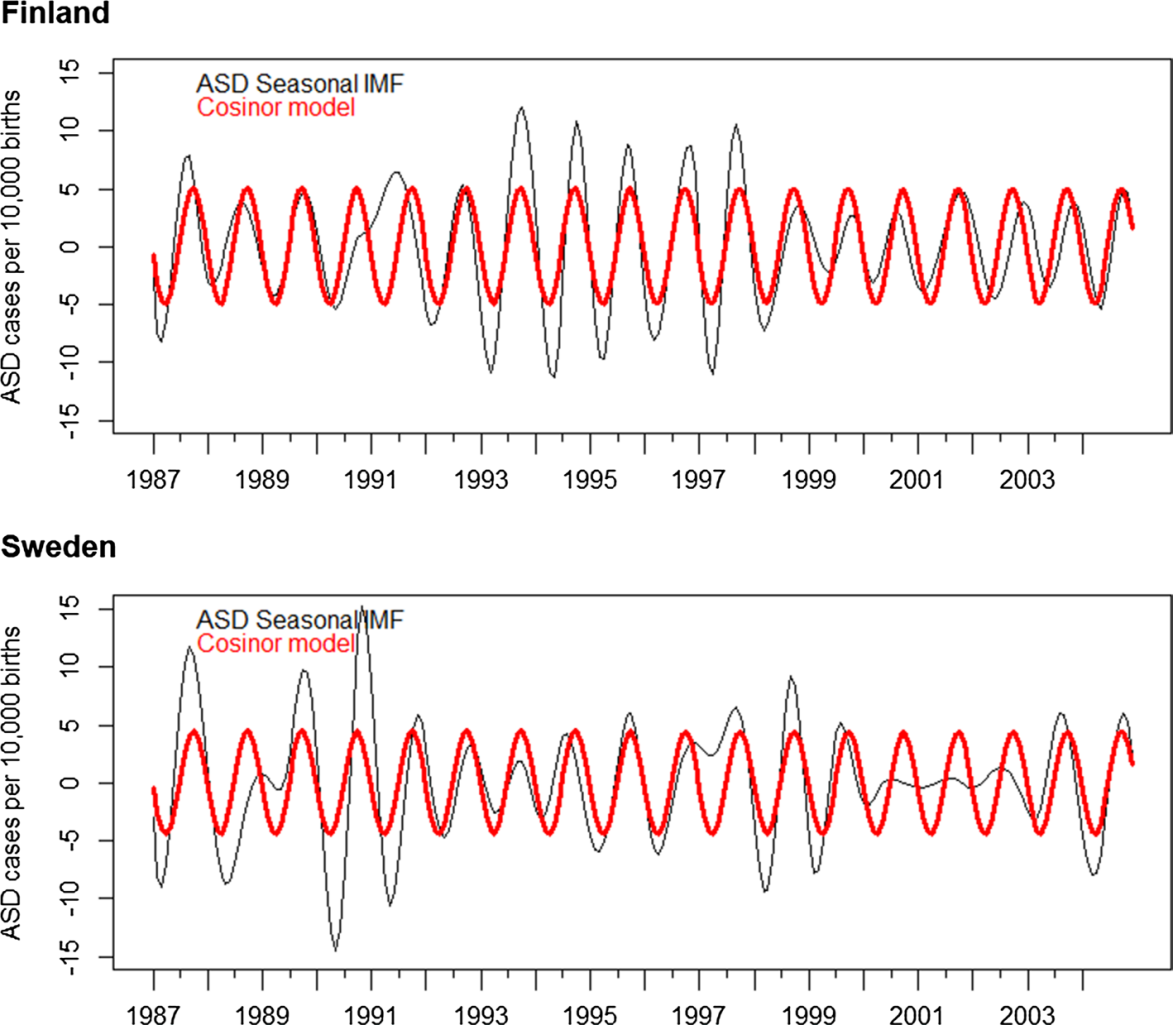
Seasonal IMFs in ASD prevalence time series and fitted cosinor models

We then performed EMD to decompose the ASD prevalence time series and examined the component signals (Fig. 2). Permutation testing indicated the following IMFs were significantly different from random noise: Denmark—IMF 5 and the residue; Finland—IMFs 3-5 and the residue; Norway—IMF 6 and the residue; Sweden—IMFs 3, 5, and the residue; W. Australia—IMFs 4, 5, and the residue. Of these IMFs, IMFs 5, 6, and the residue were clearly part of aforementioned calendar trends (Supplement Figures 1–5). Of the remaining statistically significant IMFs, IMFs 3 for both Finland and Sweden exhibited periods of approximately 1 year in length, consistent with the presence of yearly seasonal component for these countries. There was no support of similar seasonal components for Denmark, Norway, or Australia.

Next, we fitted cosinor models with a defined period of 1 year to IMFs 3 of the ASD time series for Finland and Sweden. These independent cosinor models were similar with each other. For Finland: β_0_ = 0.06, β_1_ = − 0.75, β_2_ = − 4.99, while for Sweden: β_0_ = 0.03, β_1_ = − 0.39, β_2_ = − 4.46. (Figure 2). Using the fitted cosinor models, we estimated the excess cases that were attributable to seasonal trends for these countries. Estimates of excess rates were consistent with the general pattern of the odds ratios estimated from logistic regression, in finding excess cases occurring in the latter months of the year (Table 2). The peak excess was observed for children born in the month of October: 5.1 and 4.5 extra ASD cases per 10,000 for Finland and Sweden, respectively, while the lowest rates were observed for the birth month of April, with 5.0 and 4.4 fewer ASD cases per 10,000 births for Finland and Sweden, respectively.

**Table 2:** Difference in number of ASD cases per 10,000 births by birth month attributable to seasonal variation (estimate and 95% confidence interval)

	Finland	Sweden
Jan	− 0.7 (− 1.5, 0.1)	− 0.4 (− 1.2, 0.5)
Feb	− 3.1 (− 3.9, − 2.3)	− 2.6 (− 3.5, − 1.7)
Mar	− 4.6 (− 5.4, − 3.8)	− 4.0 (− 4.9, − 3.1)
Apr	− 5.0 (− 5.7, − 4.2)	− 4.4 (− 5.3, − 3.5)
May	− 4.0 (− 4.8, − 3.2)	− 3.7 (− 4.6, − 2.8)
Jun	− 1.9 (− 2.7, − 1.1)	− 1.9 (− 2.8, − 1.0)
Jul	0.7 (− 0.1, 1.5)	0.3 (− 0.6, 1.2)
Aug	3.1 (2.4, 3.9)	2.5 (1.7, 3.4)
Sep	4.7 (4.0, 5.5)	4.1 (3.2, 5.0)
Oct	5.1 (4.3, 5.8)	4.5 (3.6, 5.4)
Nov	4.0 (3.2, 4.8)	3.7 (2.8, 4.6)
Dec	2.0 (1.2, 2.7)	2.0 (1.1, 2.9)

**Fig. 3:**
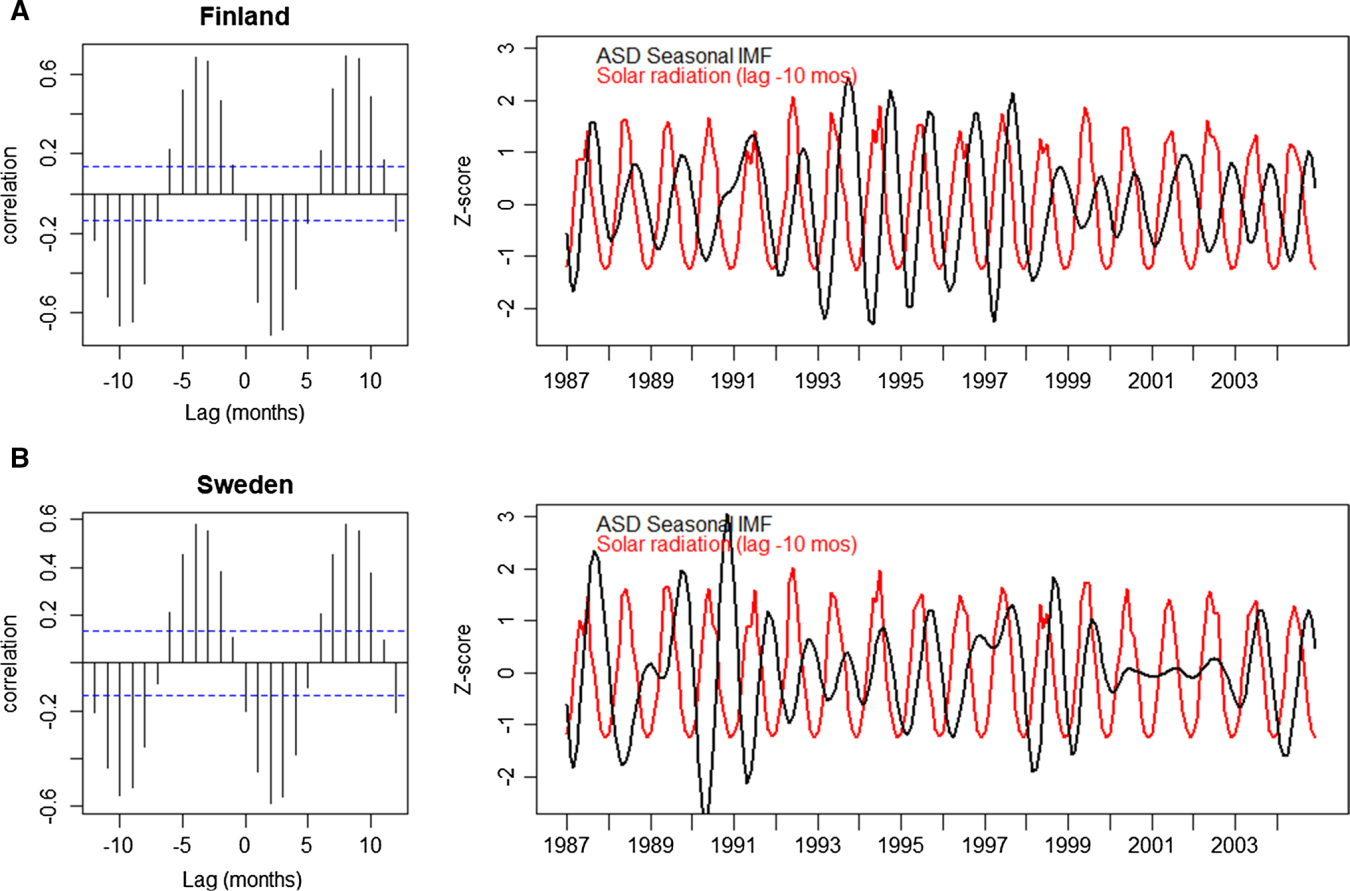
Cross correlation functions and lagged plots relating solar radiation as a predictor of seasonal ASD prevalence. The dashed blue lines represent an approximate 95% confidence interval for what is produced by white noise

We next examined the cross-correlations between these seasonal IMFs and incident solar radiation. The largest inverse correlations were seen with lags of − 10 months, i.e., around conception (Finland: − 0.67; Sweden: − 0.55) and + 2 months, i.e., 2 months after delivery (Finland: − 0.71; Sweden: − 0.59) (Fig. 3). Linear regression of the seasonal IMFs and solar radiation with a lag of − 10 months yielded adjusted R^2^ values of 0.49 and 0.35 for Finland and Sweden, respectively. Corresponding adjusted R^2^ values for lag + 2 months were 0.50 and 0.34. Thus, changes in solar radiation 10 months prior or 2 months after birth explained approximately one-third to one-half of detected seasonal trends in ASD prevalence.

## Discussion

We found evidence supporting the presence of seasonal trends in Finland and Sweden, with a modest increase in ASD risk for births in the fall months (i.e., conceived in the winter), and the lowest risk for births in the spring months (i.e., conceived in the summer). The peak in ASD cases was observed for the birth month of October while the trough was observed for April. Strong evidence of seasonality in ASD births was found for Finland and Sweden, but not for Denmark, Norway, or Australia. It is possible that background noise for these countries was too strong to extract the same seasonal signals that were detected in Finland and Sweden. This ‘noise’—in other words, any other influence on ASD prevalence rates—could be composed of multiple causes, such as sudden changes in diagnostic criteria or reporting practices, or changes in other risk factors. Additional factors that may have influenced results could be country-specific. For Norway, the reported prevalence of ASD was low and predominantly childhood autism, which reduced statistical power. For Australia, if sunlight is a factor contributing to seasonal effects, it may be possible that sunlight levels are generally high and do not fall below a threshold that would induce variability in ASD risk. The finding of no seasonality for Denmark is consistent with a prior study [12].

The present findings of higher ASD prevalence for fall births and lower prevalence for spring births is difficult to compare against other seasonality studies of ASD and for other disorders such as schizophrenia, bipolar disorder, and major depressive disorder, since earlier studies did not use signal decomposition methods to determine seasonal patterns. We note that even while our logistic regression analysis adjusted for birth year, any potential seasonal signals were contaminated by background noise as demonstrated by the EMD analysis. Such contamination would likely be present also in other seasonality studies. In general, we would expect that empirical mode decomposition, which explicitly extracted signals while removing noise from the data, would perform more capably in noisy data situations than methods that merely attempted to adjust for such components.

Our study has a number of strengths. First, we had the opportunity to compare seasonal trends across multiple countries. Although we were not able to detect seasonality in all five countries examined, the similarity of observed trends for both Finland and Sweden supported the existence of a common seasonal component to ASD prevalence, reducing the likelihood that this finding was due to chance. Second, the inclusion of multiple years of birth cohorts allowed for the detection of a long-term stationary seasonal trend that did not change from year to year, thus providing greater confidence that detected seasonal trends were not just a chance occurrence. Finally, the use of both parametric and non-parametric methods to decompose the ASD data represents a significant methodological advance in the study of seasonality of ASD.

There were some limitations with the analysis. First, some studies have suggested that seasonal effects on developmental outcomes may be at least partially attributable to non-causal factors such as socioeconomic status or maternal intelligence [31]. The EMD analyses were performed on aggregated time series data and thus could not take into account such covariates. However, we performed a sensitivity analysis for the Swedish data, where we had access to data on maternal education. Log odds estimates for each month differed on average by 4% (Supplement Table 2). This suggests that confounding by such factors was not likely to explain the observed seasonal trends. Interestingly, a recent GWAS study of schizophrenia arrived at a similar conclusion in determining that any seasonality effect was likely due to a pathogenic environmental exposure [32] Second, EMD decomposes time series into IMFs which may be subjectively interpreted. We addressed this limitation by applying stringent statistical thresholds to identify only the most likely signals, thus reducing the risk of false positives. We also used cosinor modeling to determine that candidate signals were consistent with what would be expected from seasonal trends. This parametric method helped provide eye-test assurance that identified seasonal signals were indeed valid. Another limitation is that sunlight in the capital cities of Helsinki and Stockholm was used as proxies for sunlight exposure across the entire countries of Finland and Sweden.

Sunlight may play a role in the mechanism underlying seasonality. Our analyses indicated inverse correlations between sunlight levels around the time of conception and in the postnatal period and ASD prevalence. This is consistent with recent studies suggesting that low maternal levels of the photodependent vitamin D may be associated with increased risk of ASD in the offspring [33–35]. However, other causal factors, including latitude, diet and dietary supplements, and behaviors, might also affect in utero vitamin D exposure. In addition, several unrelated causal factors, such as maternal viral infections and particulate matter air pollution, might also contribute to the presence of seasonal trends.

## Conclusion

In one of the largest analyses of ASD birth seasonality and the first multinational study to date, there was evidence supporting the presence of seasonal trends in Finland and Sweden, but not for Denmark, Norway, and Western Australia. The highest risk was observed for fall births and the lowest risk for spring births. Assuming that season of birth is a proxy for temporally fluctuating environmental conditions, this study provides further support of the involvement of non-genetic risk factors in the etiology of ASD.

## Electronic supplementary material

Below is the link to the electronic supplementary material.

Supplementary material 1 (DOCX 447 kb)
