# Supplementary material for: Birth seasonality and risk of autism spectrum disorder
Source: Eur J Epidemiol. 2019 Mar 20;34(8):785–92. doi: 10.1007/s10654-019-00506-5 (PMC6602987; doi:10.1007/s10654-019-00506-5)
Supplement: Supplementary file 1 — Supplementary material 1 (DOCX 447 kb) [file 10654_2019_506_MOESM1_ESM.docx]

**Supplement Table 1**: Aggregate totals of country-specific ASD prevalences by birth month. N, ASD | N, total births (ASD rate per 10,000)

|  | Denmark | Finland | Norway | Sweden | Australia |
| --- | --- | --- | --- | --- | --- |
|  | 1987-2004 | 1987-2004 | 1987-2004 | 1987-2004 | 1987-1999 |
| Jan | 1037\|94054 (1.1) | 566\|89401 (0.63) | 112\|87369 (0.13) | 1127\|153097 (0.74) | 88\|25210 (0.35) |
| Feb | 1072\|89623 (1.2) | 536\|84269 (0.64) | 80\|83944 (0.1) | 1118\|150290 (0.74) | 83\|24214 (0.34) |
| Mar | 1096\|100618 (1.09) | 609\|97275 (0.63) | 112\|94067 (0.12) | 1267\|170680 (0.74) | 88\|26746 (0.33) |
| Apr | 1091\|99675 (1.09) | 642\|94676 (0.68) | 97\|93757 (0.1) | 1225\|168876 (0.73) | 105\|25634 (0.41) |
| May | 1133\|100982 (1.12) | 594\|94197 (0.63) | 122\|93411 (0.13) | 1304\|165897 (0.79) | 92\|26228 (0.35) |
| Jun | 1173\|100015 (1.17) | 589\|92430 (0.64) | 116\|89654 (0.13) | 1176\|158544 (0.74) | 83\|25567 (0.32) |
| Jul | 1209\|105970 (1.14) | 690\|95909 (0.72) | 121\|93072 (0.13) | 1316\|162050 (0.81) | 96\|26003 (0.37) |
| Aug | 1157\|103925 (1.11) | 648\|93694 (0.69) | 101\|89785 (0.11) | 1188\|156830 (0.76) | 113\|25652 (0.44) |
| Sep | 1227\|100205 (1.22) | 644\|91413 (0.7) | 110\|88548 (0.12) | 1254\|150079 (0.84) | 106\|26463 (0.4) |
| Oct | 1141\|96627 (1.18) | 667\|87615 (0.76) | 101\|84704 (0.12) | 1215\|143701 (0.85) | 90\|26072 (0.35) |
| Nov | 1060\|90309 (1.17) | 577\|82391 (0.7) | 98\|78958 (0.12) | 1146\|130018 (0.88) | 101\|23751 (0.43) |
| Dec | 1031\|90513 (1.14) | 626\|84557 (0.74) | 114\|80309 (0.14) | 1165\|131130 (0.89) | 89\|23975 (0.37) |
|  |  |  |  |  |  |
| TOTAL | 13427\|1172516 (1.15) | 7388\|1087827 (0.68) | 1284\|1057578 (0.12) | 14501\|1841192 (0.79) | 1134\|305515 (0.37) |

**Supplement** **Table 2**: Percent different in log odds estimates for association of birth month and ASD in Sweden adjusted for maternal education

|  | % difference |
| --- | --- |
| Jan | - |
| Feb | 12.9 |
| Mar | 13.0 |
| Apr | 9.7 |
| May | 5.6 |
| Jun | 2.4 |
| Jul | 0.8 |
| Aug | 0.9 |
| Sep | -0.1 |
| Oct | 0.4 |
| Nov | -1.0 |
| Dec | -1.1 |

**Supplement Figure 1**: Empirical mode decomposition of Denmark ASD prevalence time series

| **A**  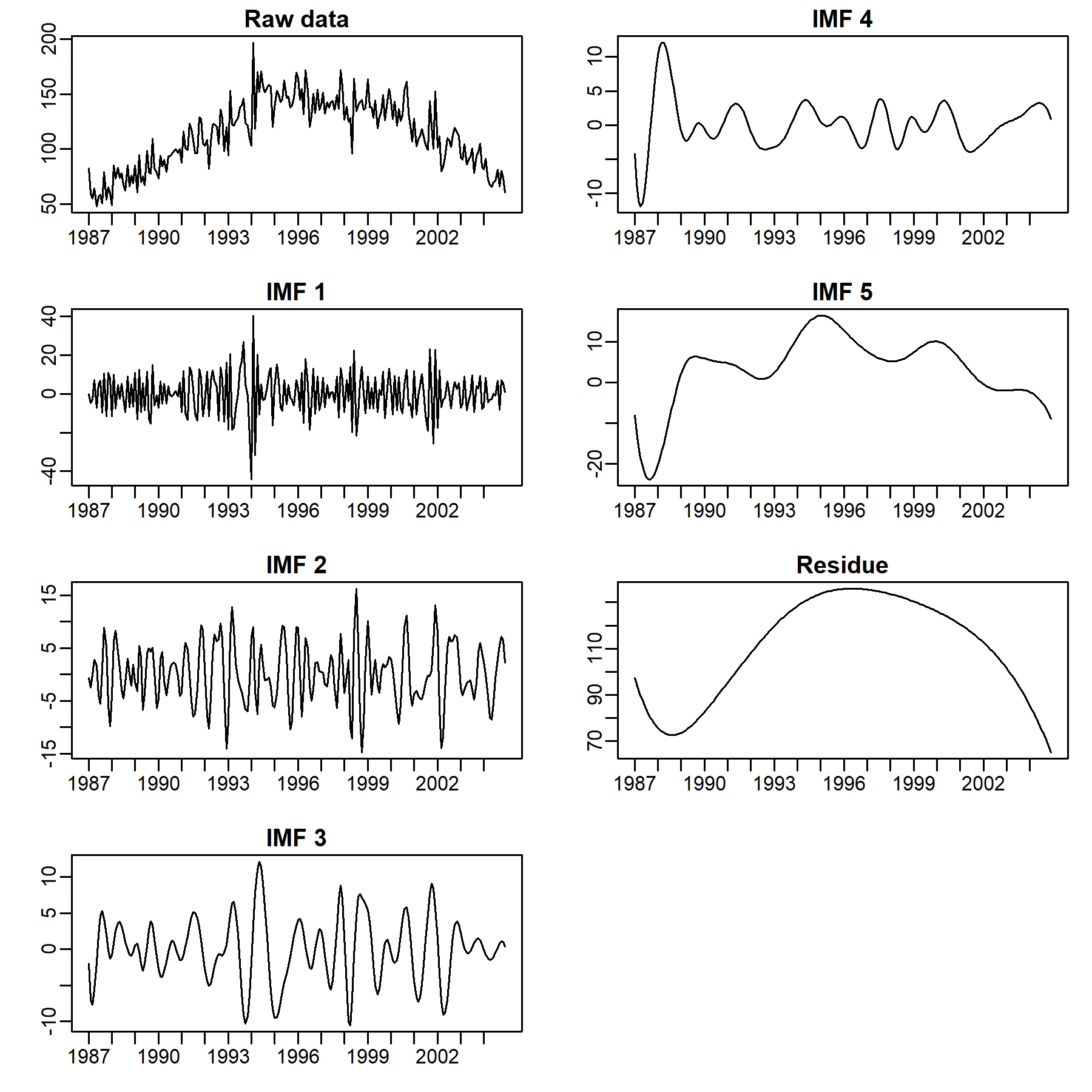 | |
| --- | --- |
| **B**  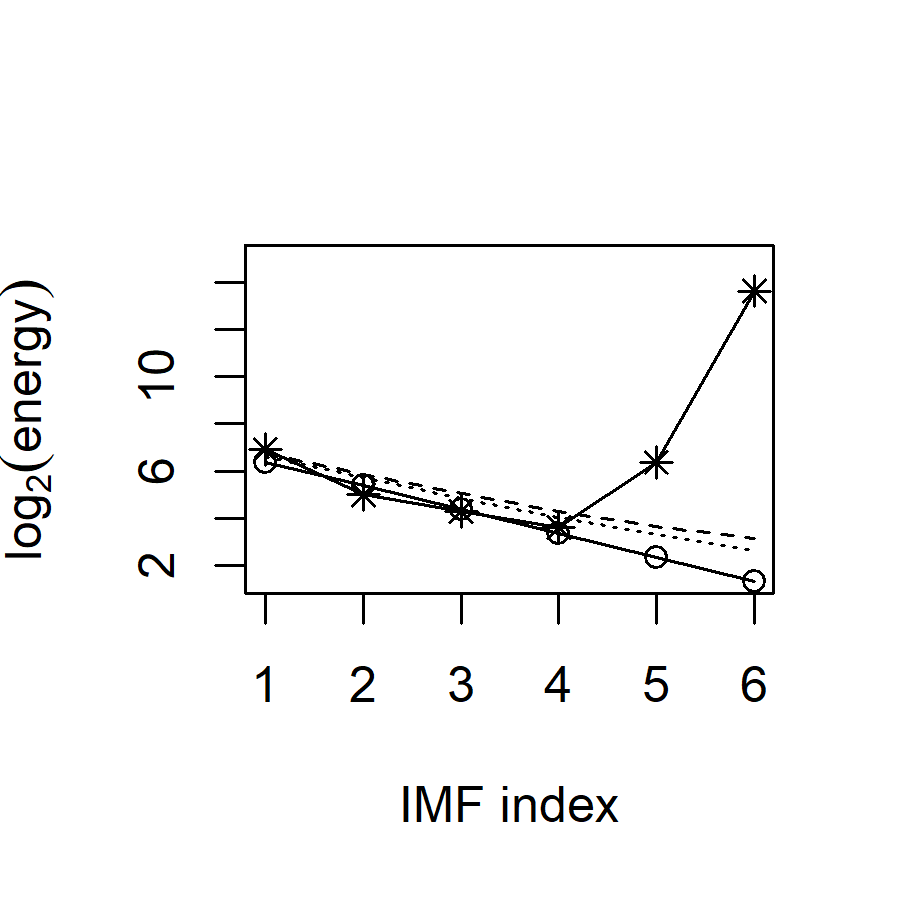 | **C**  **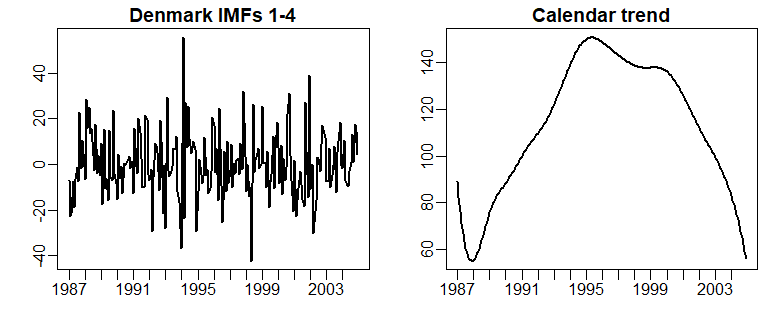** |

ASD: Autism Spectrum Disorder, IMF: Intrinsic Mode Function.

**Supplement Figure 2:** Empirical mode decomposition of Finland ASD prevalence time series

| **A**  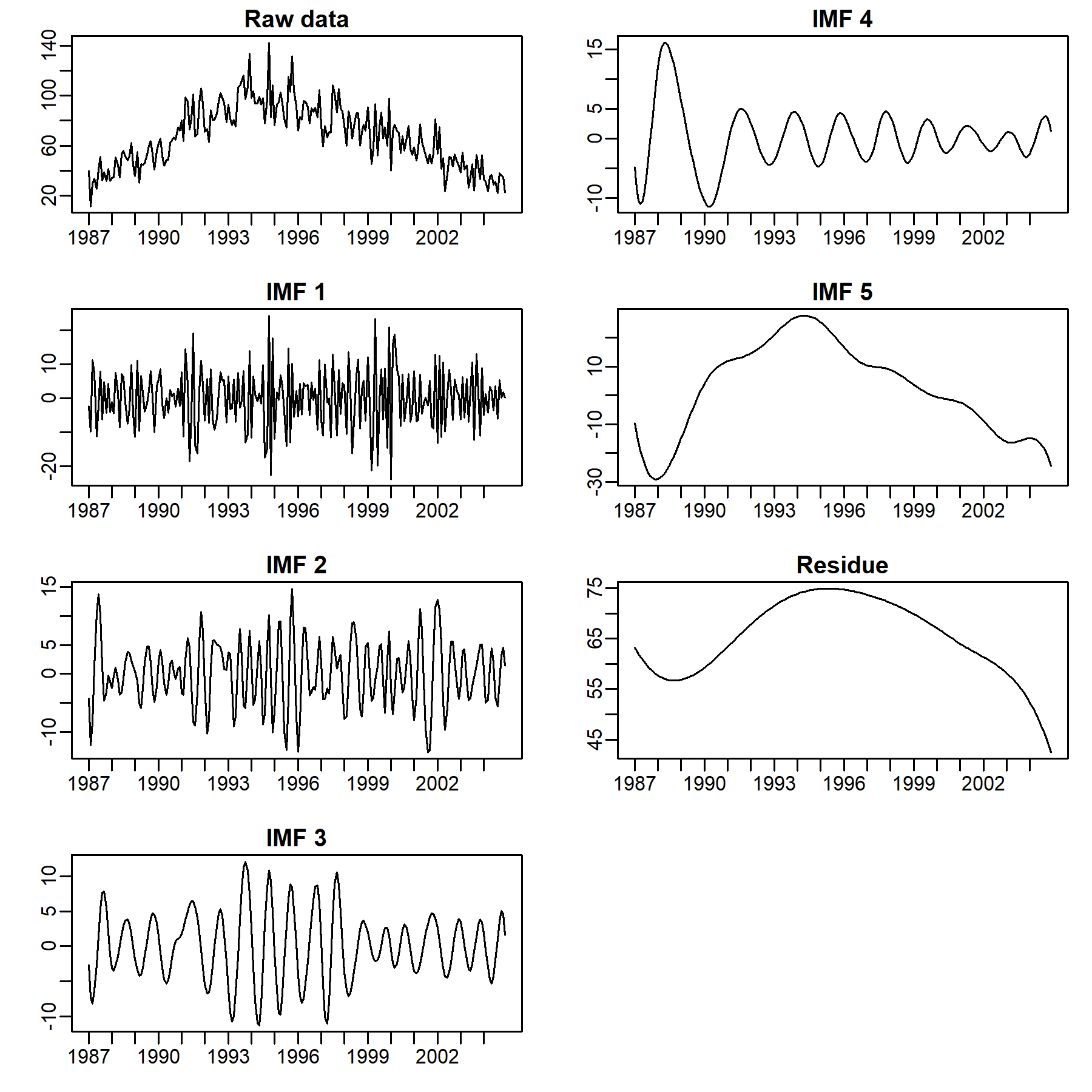 | |
| --- | --- |
| **B**  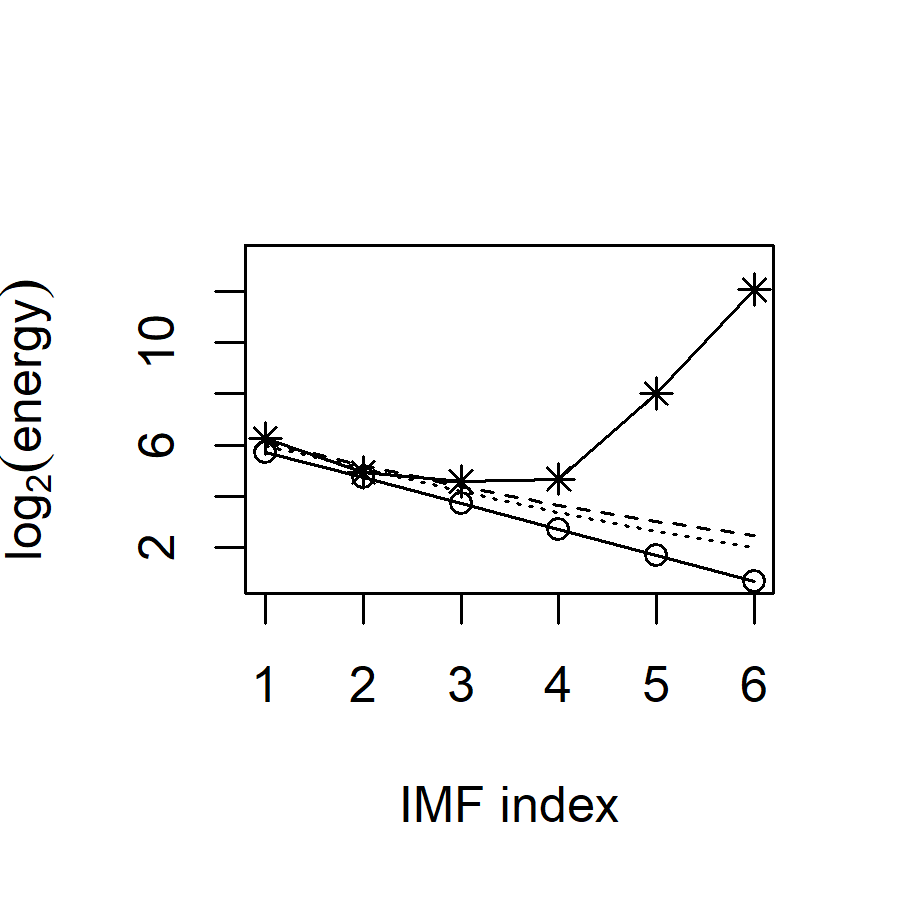 | **C**  **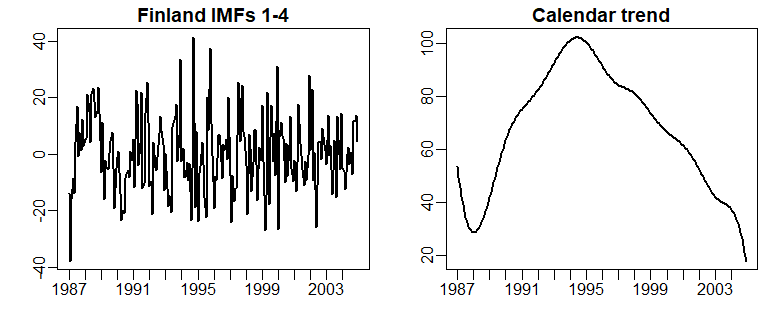** |

ASD: Autism Spectrum Disorder, IMF: Intrinsic Mode Function.

**Supplement Figure 3**: Empirical mode decomposition of Norway ASD prevalence time series

| **A**  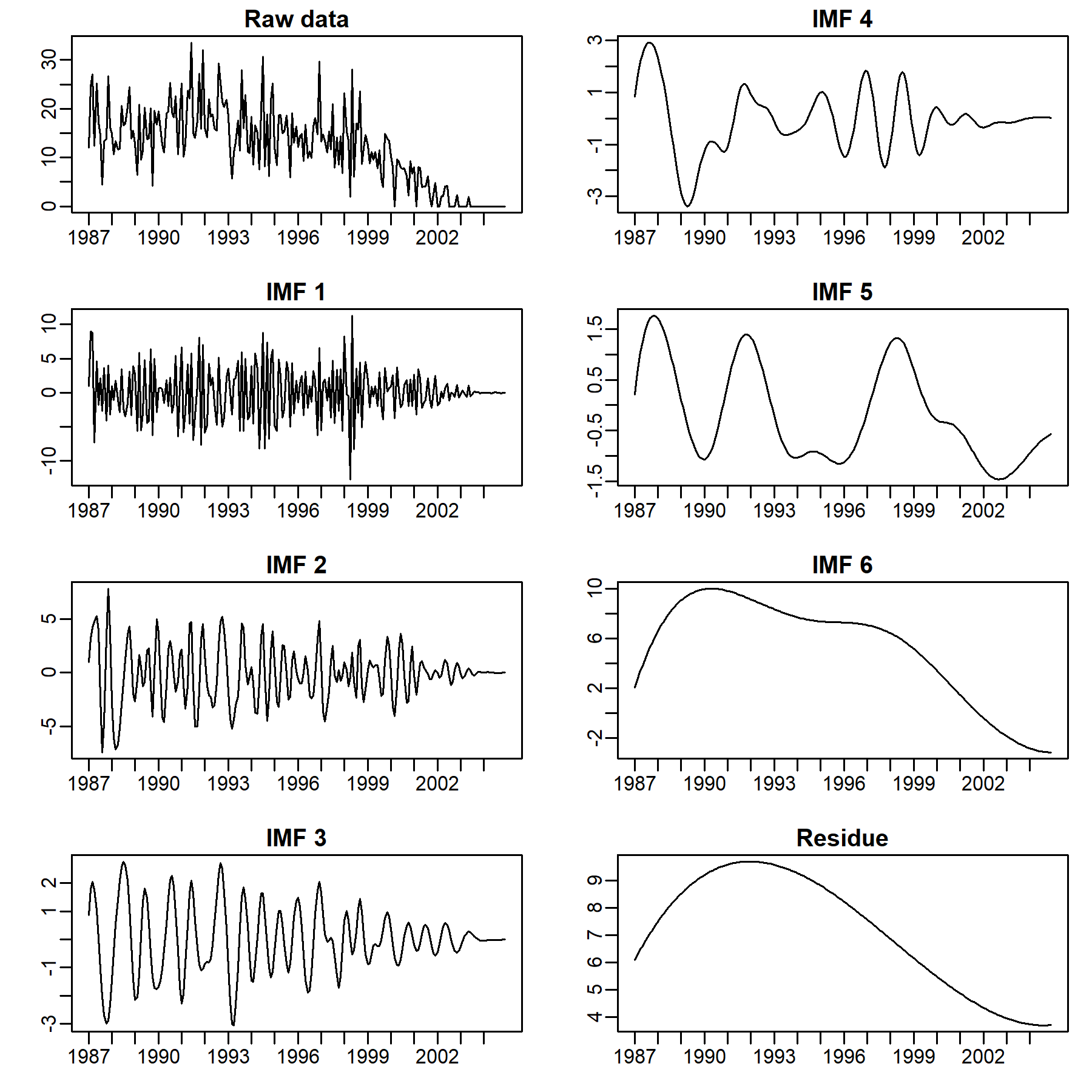 | |
| --- | --- |
| **B**  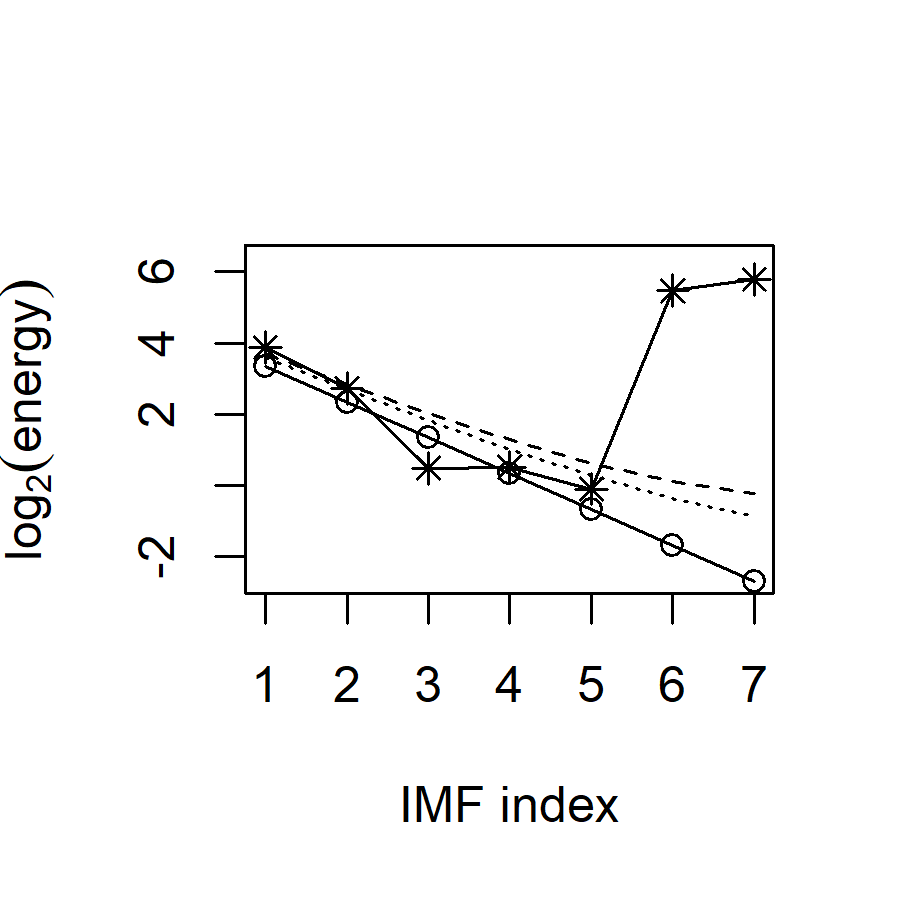 | **C**  **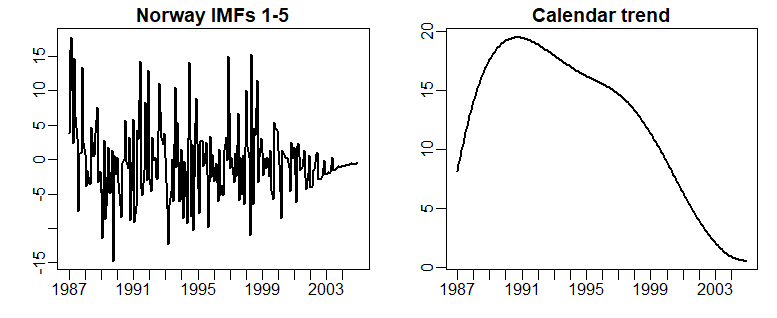** |

ASD: Autism Spectrum Disorder, IMF: Intrinsic Mode Function.

**Supplement Figure 4**: Empirical mode decomposition of Sweden ASD prevalence time series

| **A**  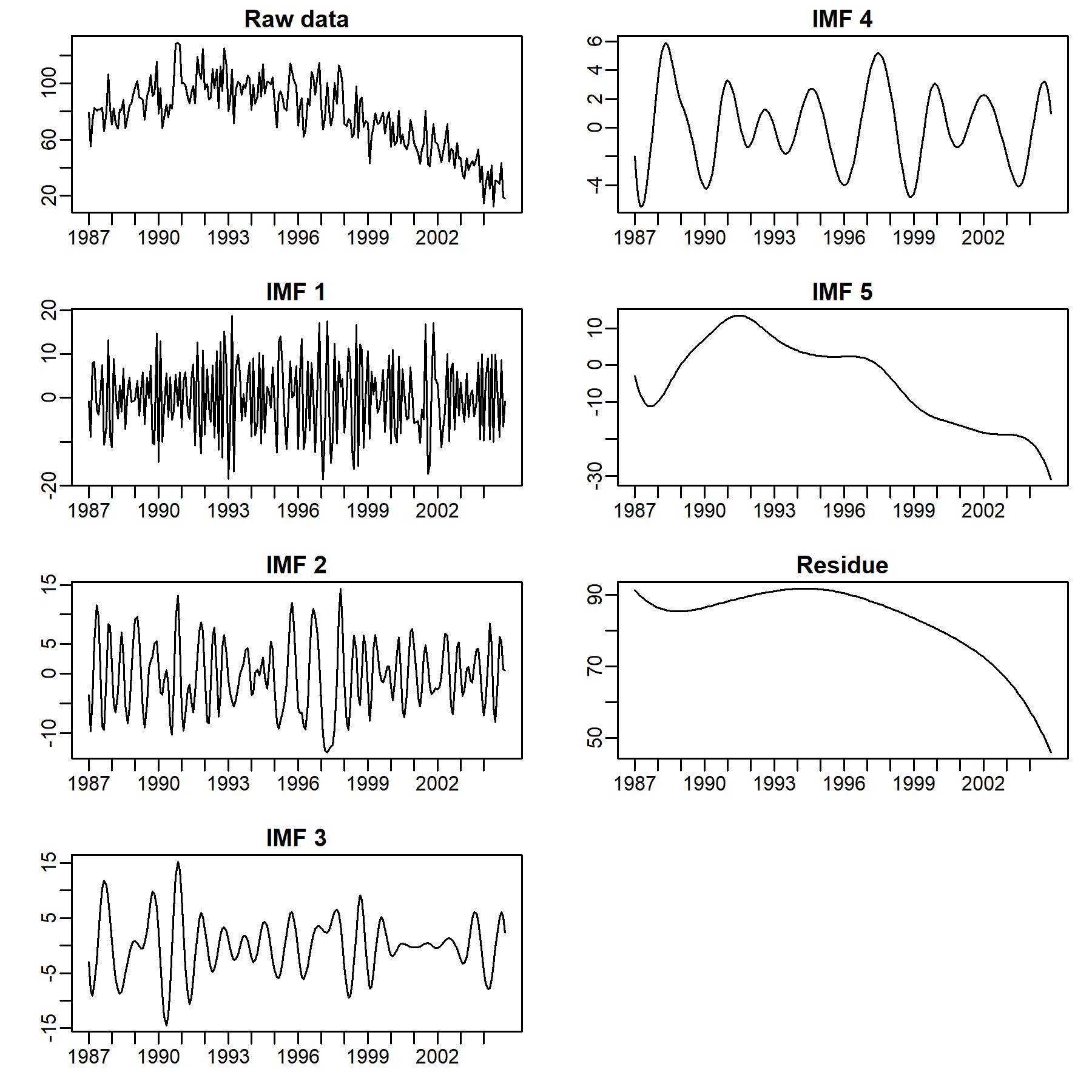 | |
| --- | --- |
| **B**  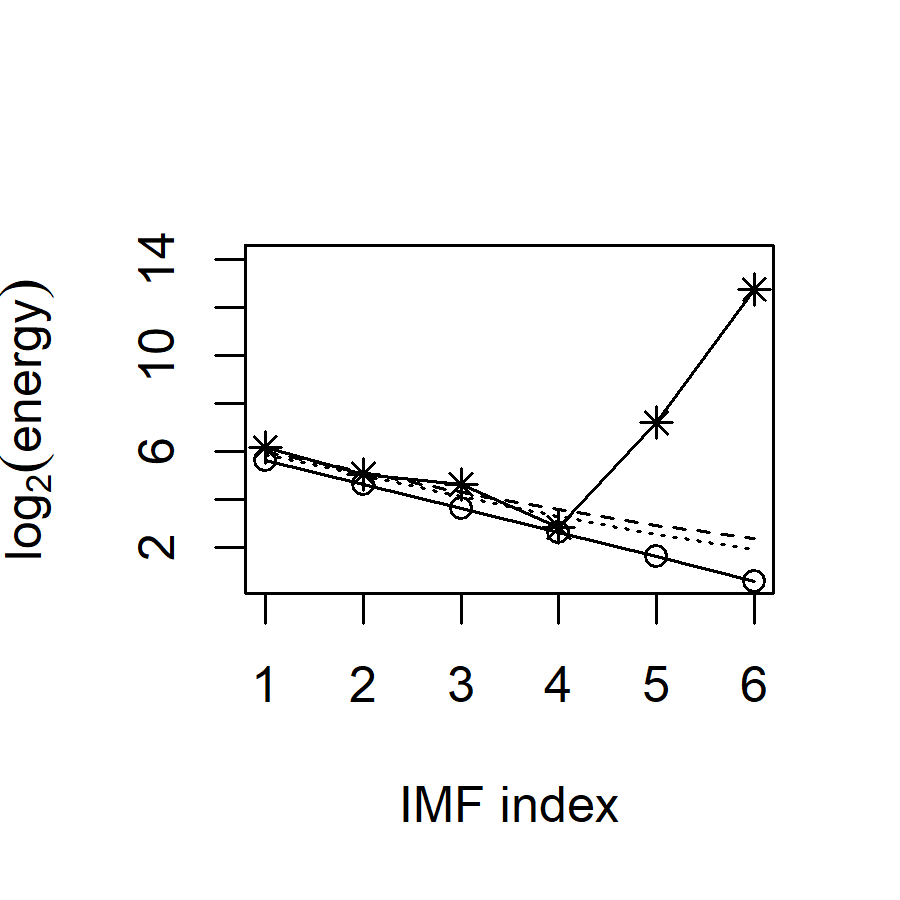 | **C**  **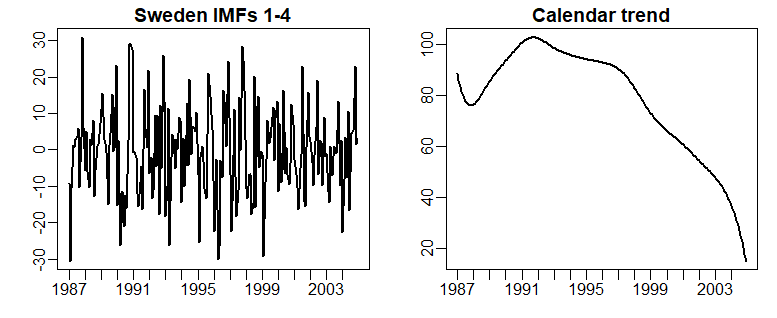** |

ASD: Autism Spectrum Disorder, IMF: Intrinsic Mode Function.

**Supplement Figure 5**: Empirical mode decomposition of Western Australia ASD prevalence time series

| **A**  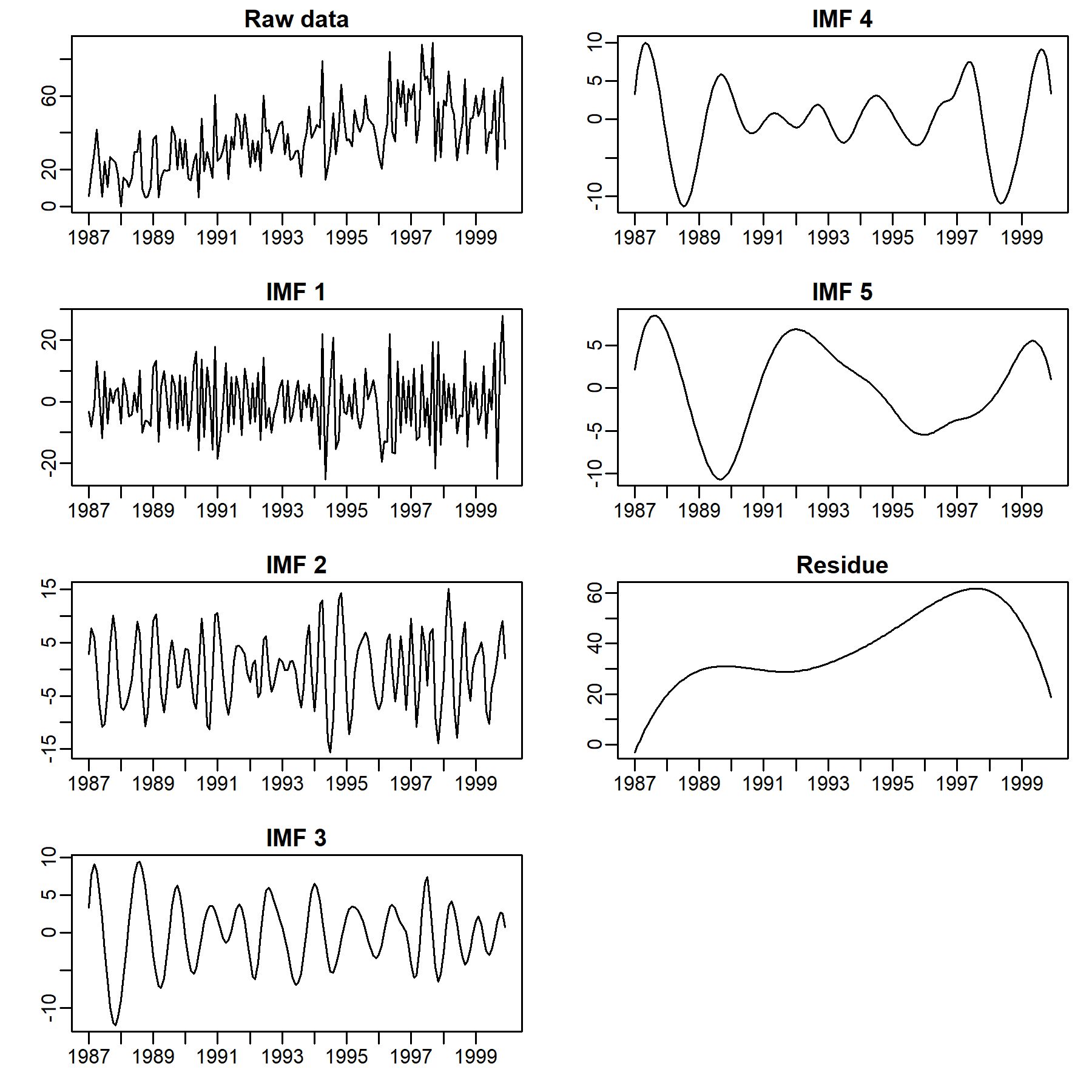 | |
| --- | --- |
| **B**  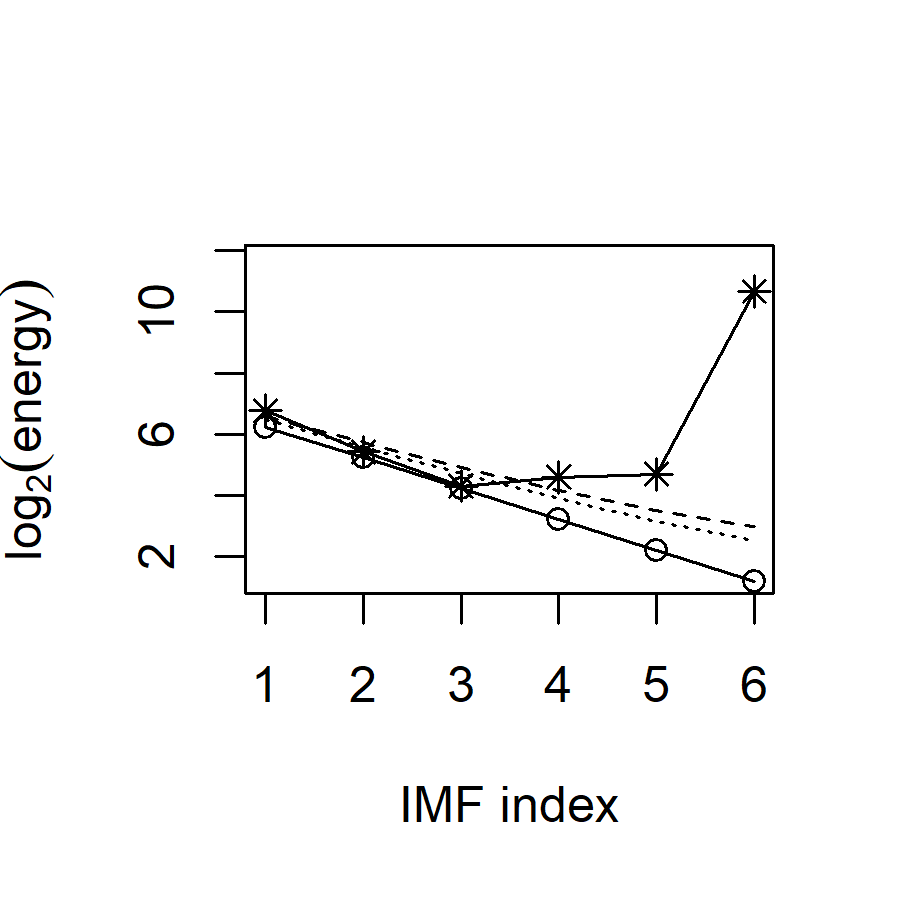 | **C**  **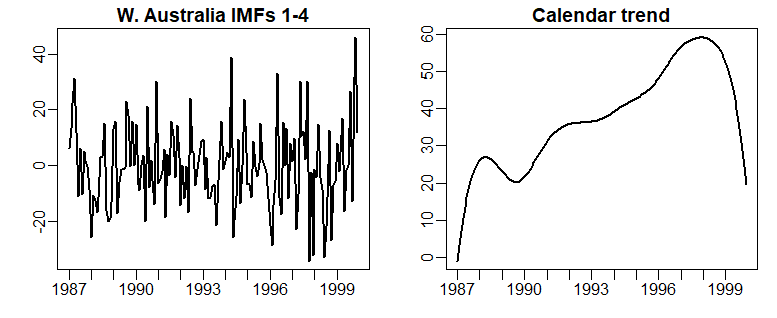** |

ASD: Autism Spectrum Disorder, IMF: Intrinsic Mode Function.
